# Supplementary figures and images for: Characterization of the microbiome of the invasive Asian toad in Madagascar across the expansion range and comparison with a native co-occurring species
Source: PeerJ. 2021 Jun 28;9:e11532. doi: 10.7717/peerj.11532 (PMC8247705; doi:10.7717/peerj.11532)

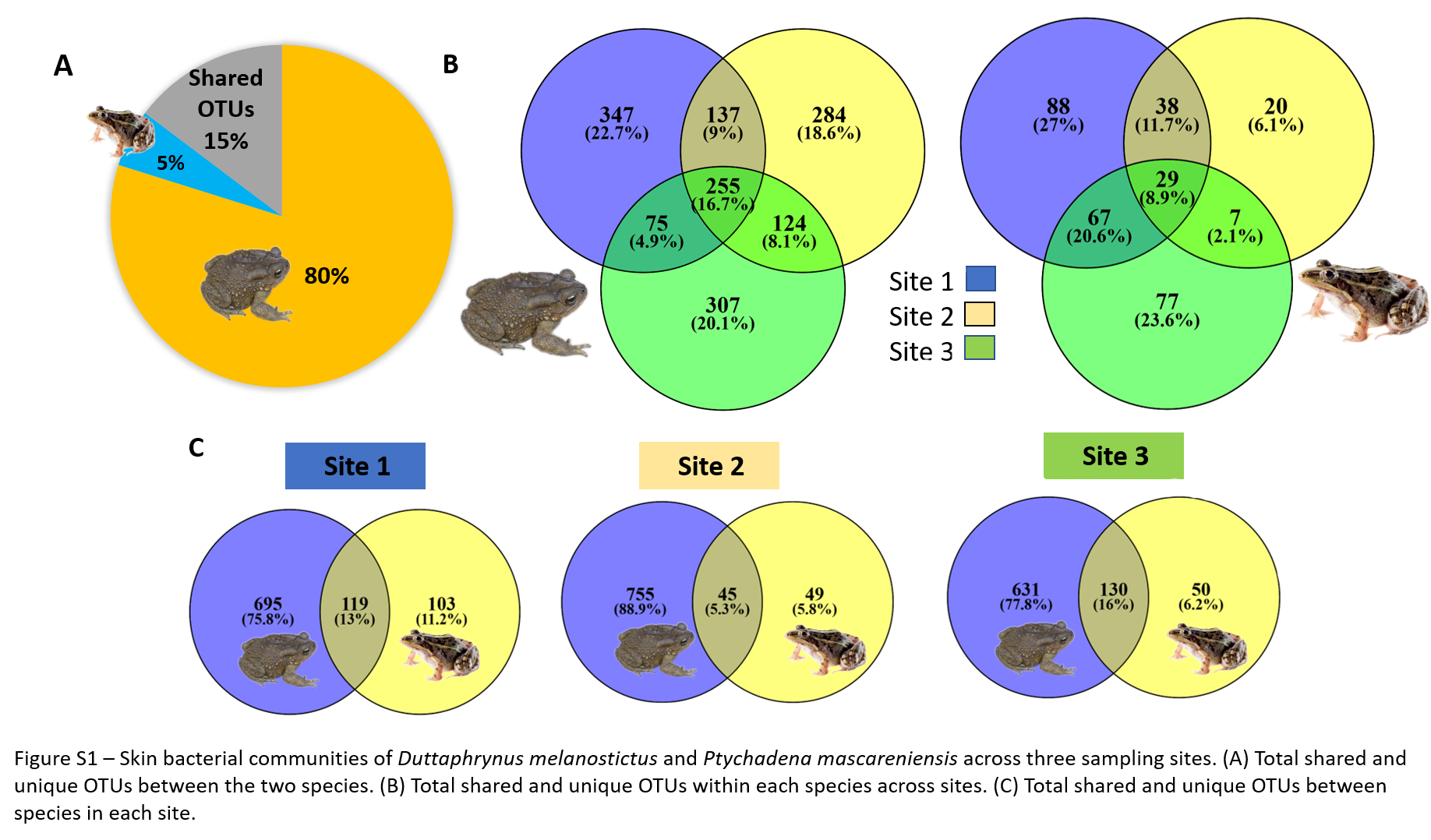

Supplement: Supplemental Information 1 — (A) Total shared and unique OTUs between the two species. (B) Total shared and unique OTUs within each species across sites. (C) Total shared and unique OTUs between species in each site. Photo credit: Angelica Crottini, Javier Lobon-Rovira. [file peerj-09-11532-s001.png]

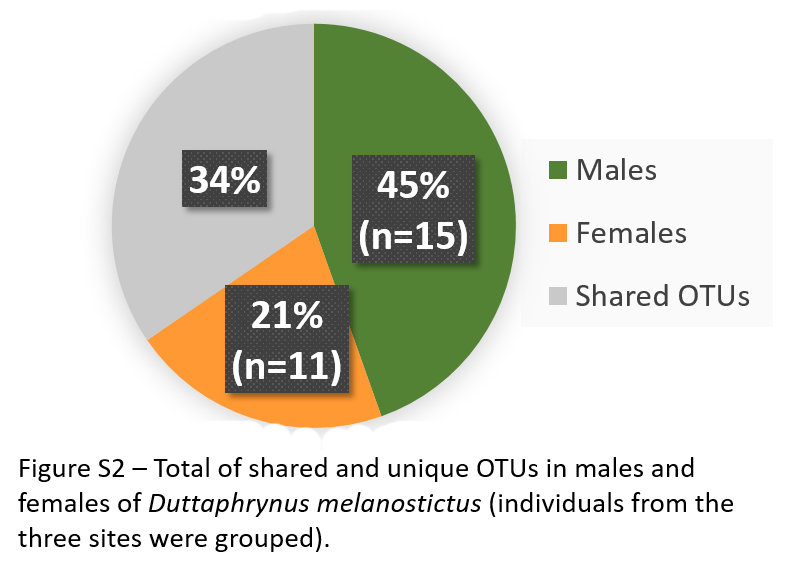

Supplement: Supplemental Information 2 [file peerj-09-11532-s002.png]

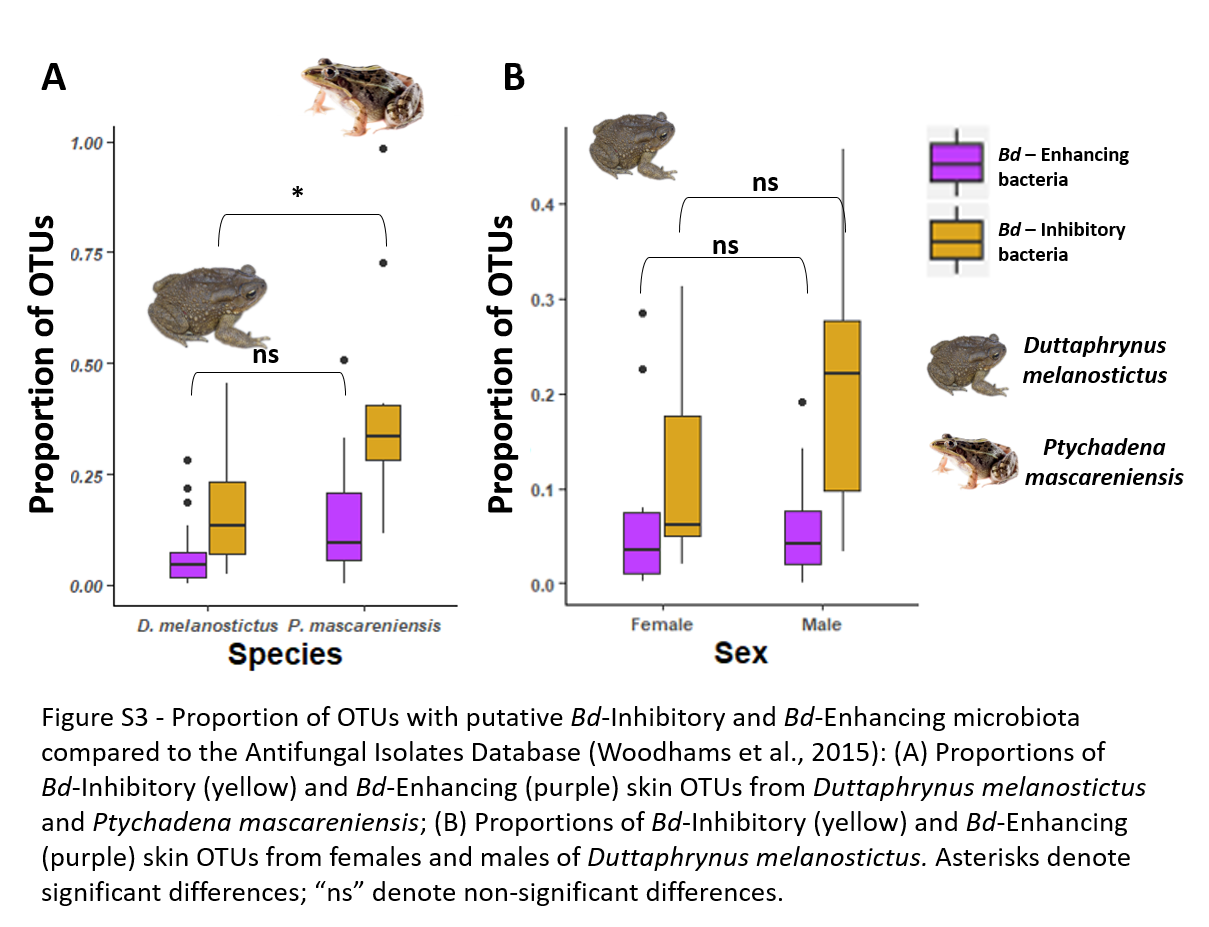

Supplement: Supplemental Information 3 — (A) Proportions of Bd-Inhibitory (yellow) and Bd-Enhancing (purple) skin OTUs from Duttaphrynus melanostictus and Ptychadena mascareniensis; (B) Proportions of Bd-Inhibitory (yellow) and Bd-Enhancing (purple) skin OTUs from females and males of Duttaphrynus melanostictus. Asterisks denote significant differences; “ns” denote non-significant differences Photo credit: Angelica Crottini, Javier Lobon-Rovira. [file peerj-09-11532-s003.png]

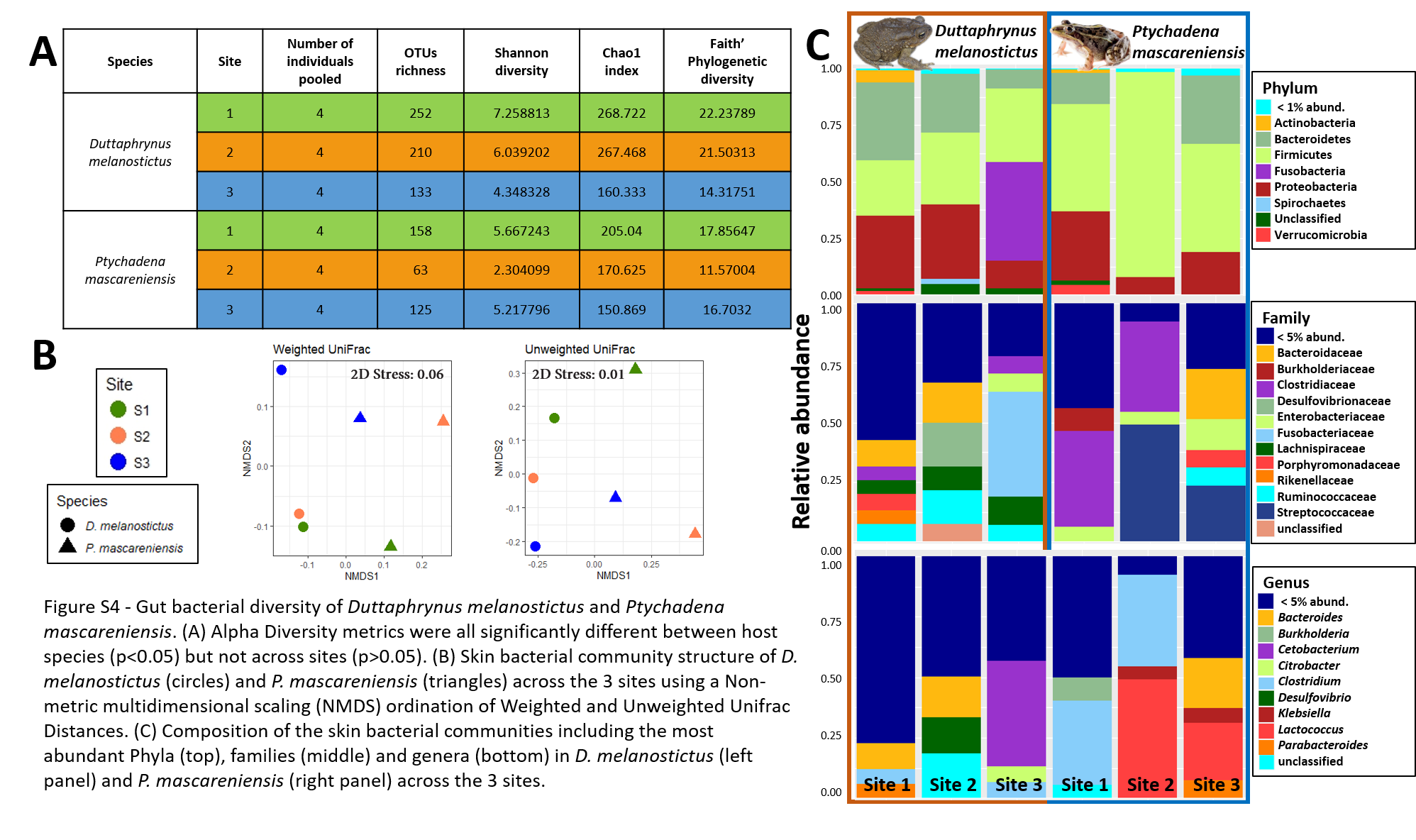

Supplement: Supplemental Information 4 — A-D) Alpha Diversity metrics were all significantly different between host species (p < 0.05) but not across sites (p > 0.05); E–F) Skin bacterial community structure of D. melanostictus (circles) and P. mascareniensis (triangles) across the 3 sites using a Non-metric multidimensional scaling (NMDS) ordination of Weighted and Unweighted Unifrac Distances; G–H) Composition of the skin bacterial communities including the most abundant Phyla (top), families (middle) and genera (bottom) in D. melanostictus (left panel) and P. mascareniensis (right panel) across the 3 sites. Photo credit: Angelica Crottini, Javier Lobón-Rovira. [file peerj-09-11532-s004.png]

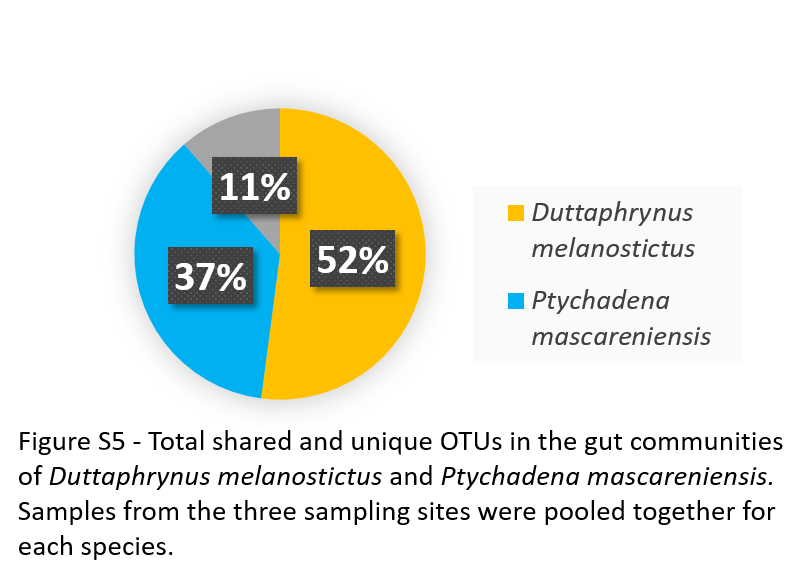

Supplement: Supplemental Information 5 [file peerj-09-11532-s005.png]
