# Supplementary material for: Characterization of the microbiome of the invasive Asian toad in Madagascar across the expansion range and comparison with a native co-occurring species
Source: PeerJ. 2021 Jun 28;9:e11532. doi: 10.7717/peerj.11532 (PMC8247705; doi:10.7717/peerj.11532)
Supplement: Supplemental Information 6 [file peerj-09-11532-s006.docx]

**Table S1:**

**Total number of samples, sequences and OTUs available in each dataset before and after each filter.**

|  | Initial values | | OTUs filtering (OTUS with <0.001% of total reads removed) | | Rarefaction Level | Values after Rarefaction | | |
| --- | --- | --- | --- | --- | --- | --- | --- | --- |
|  | **Total of sequences** | **Total of samples** | **Total of sequences** | **Total of samples** | **Rarefaction Level** | **Total of sequences** | **Number of OTUs**  **(Deblur)** | **Total of samples** |
| Skin swabs  (Datasets A and B) | 576,721 | 37 | 575,256 | 37 | 1,455/4,000 | 53,835/132,000 | 1,617/1,829 | 37/33 |
| Gut tissue (Dataset C) | 56,941 | 6 | 56,941 | 6 | 1,867 | 11,202 | 701 | 6 |
