# Supplementary material for: Characterization of the microbiome of the invasive Asian toad in Madagascar across the expansion range and comparison with a native co-occurring species
Source: PeerJ. 2021 Jun 28;9:e11532. doi: 10.7717/peerj.11532 (PMC8247705; doi:10.7717/peerj.11532)
Supplement: Supplemental Information 7 [file peerj-09-11532-s007.docx]

**Table S2:** Samples available in datasets A, B and C after sequence processing. For dataset A, values correspond to the number of samples obtained after rarefaction (1,455 or 4,000).

| **DATASET A (Rarefaction: 1,455/4,000)** | | | **DATASET B  (Rarefaction: 1,455)** | | | **DATASET C**  **(Rarefaction: 1,867)** | | |
| --- | --- | --- | --- | --- | --- | --- | --- | --- |
| **Skin bacterial communities** | | | **Skin bacterial communities**  **(*Duttaphrynus melanostictus*)** | | | **Gut bacterial communities** | | |
| **Site** | ***Duttaphrynus melanostictus*** | ***Ptychadena mascareniensis*** | **Site** | **Males** | **Females** | **Site** | ***Duttaphrynus melanostictus*** | ***Ptychadena***  ***mascareniensis*** |
| **S1** | 9/9 | 4/3 | **S1** | 3 | 6 | **S1** | Pool of 4 individuals | Pool of 4 individuals |
| **S2** | 10/9 | 3/2 | **S2** | 6 | 4 | **S2** | Pool of 4 individuals | Pool of 4 individuals |
| **S3** | 7/6 | 4/4 | **S3** | 6 | 1 **(a)** | **S3** | Pool of 4 individuals | Pool of 4 individuals |
| **Total of samples** | 26/24 | 11/9 | **Total of samples** | 15 | 11 | **Total of samples** | 3 pools | 3 pools |

**Notes: (a)** At site 3, only 1 female was collected, so this was excluded from the statistical analysis.
