# Supplementary material for: Characterization of the microbiome of the invasive Asian toad in Madagascar across the expansion range and comparison with a native co-occurring species
Source: PeerJ. 2021 Jun 28;9:e11532. doi: 10.7717/peerj.11532 (PMC8247705; doi:10.7717/peerj.11532)
Supplement: Supplemental Information 8 — Groups that present significant difference (Kruskal-Wallis test) in the abundance levels between host species are colored. In yellow are the groups that were more abundant in D. melanostictus and in blue the groups more abundant in P. mascareniensis. [file peerj-09-11532-s008.docx]

**Table S3:**

**Predicted abundance of KEGG ortholog groups (Level 2 KOs) from skin bacterial communities of *Duttaphrynus melanostictus* and *Ptychadena mascareniensis*.**

Groups that present significant difference (Kruskal-Wallis test) in the abundance levels between host species are colored. In yellow are the groups that were more abundant in *D. melanostictus* and in blue the groups more abundant in *P. mascareniensis****.***

| **KEGG pathways**  **(Level 2)** | ***Duttaphrynus melanostictus*** | ***Ptychadena mascareniensis*** | **% Difference (*D.melanostictus*/*P.mascareniensis*)** | **Kruskal-Wallis** | **p-value** |
| --- | --- | --- | --- | --- | --- |
| Amino Acid Metabolism | 122,871 ± 7,309 | 118,494 ± 5,958 | 3.694 | 3.714 | 0.054 |
| Biosynthesis of Other Secondary Metabolites | 10,345 ± 781 | 9,210 ± 1,406 | 12.323 | 5.106 | **0.024** |
| Cancers | 1,987 ± 319 | 1,843 ± 224 | 7.816 | 1.637 | 0.201 |
| Carbohydrate Metabolism | 120,127 ± 3,883 | 115,492 ± 7,917 | 4.013 | 2.439 | 0.118 |
| Cardiovascular Diseases | 197 ± 141 | 166 ± 78 | 19.012 | 0.000 | 1.000 |
| Cell Growth and Death | 6,713 ± 1,186 | 5,285 ± 791 | 27.013 | 13.851 | **0.000** |
| Cell Motility | 41,219 ± 10,280 | 41,297 ± 6,407 | -0.190 | 0.134 | 0.715 |
| Cellular Processes and Signaling | 49,935 ± 5,082 | 57,201 ± 7,529 | -12.703 | 7.067 | **0.008** |
| Circulatory System | 531 ± 154 | 451 ± 209 | 17.697 | 1.202 | 0.273 |
| Digestive System | 563 ± 181 | 500 ± 113 | 12.518 | 0.159 | 0.690 |
| Endocrine System | 4,305 ± 728 | 3,157 ± 819 | 36.352 | 12.175 | **0.000** |
| Energy Metabolism | 62,872 ± 2,430 | 59,216 ± 2,696 | 6.173 | 11.488 | **0.001** |
| Environmental Adaptation | 1,857 ± 402 | 1,512 ± 256 | 22.788 | 6.046 | **0.014** |
| Enzyme Families | 21,559 ± 1,071 | 23,234 ± 1,004 | -7.210 | 14.478 | **0.000** |
| Excretory System | 373 ± 123 | 339 ± 81 | 10.079 | 0.961 | 0.327 |
| Folding, Sorting and Degradation | 28,094 ± 3,007 | 27,190 ± 1,120 | 3.326 | 0.283 | 0.595 |
| Genetic Information Processing | 28,305 ± 2,338 | 27,950 ± 1,783 | 1.271 | 0.159 | 0.690 |
| Glycan Biosynthesis and Metabolism | 24,857 ± 3,384 | 24,675 ± 2,783 | 0.738 | 0.054 | 0.816 |
| Immune System | 683 ± 116 | 694 ± 106 | -1.642 | 0.216 | 0.642 |
| Immune System Diseases | 624 ± 106 | 603 ± 66 | 3.381 | 1.637 | 0.201 |
| Infectious Diseases | 5,400 ± 692 | 6,299 ± 1,031 | -14.276 | 7.067 | **0.008** |
| Lipid Metabolism | 44,600 ± 4,336 | 40,721 ± 2,386 | 9.524 | 8.746 | **0.003** |
| Membrane Transport | 150,688 ± 19,303 | 175,935 ± 16,617 | -14.351 | 10.389 | **0.001** |
| Metabolic Diseases | 844 ± 91 | 851 ± 69 | -0.793 | 0.033 | 0.855 |
| Metabolism | 33,727 ± 2,544 | 33,087 ± 3,337 | 1.934 | 0.134 | 0.715 |
| Metabolism of Cofactors and Vitamins | 49,309 ± 2,384 | 46,805 ± 1,219 | 5.350 | 8.551 | **0.003** |
| Metabolism of Other Amino Acids | 23,486 ± 1,814 | 22,916 ± 1,172 | 2.491 | 1.276 | 0.259 |
| Metabolism of Terpenoids and Polyketides | 24,216 ± 2,527 | 21,535 ± 1,374 | 12.449 | 10.389 | **0.001** |
| Nervous System | 884 ± 185 | 1,159 ± 411 | -23.727 | 5.106 | **0.024** |
| Neurodegenerative Diseases | 4,001 ± 719 | 3,812 ± 770 | 4.942 | 0.040 | 0.842 |
| Nucleotide Metabolism | 37,513 ± 1,925 | 36,121 ± 2,759 | 3.855 | 1.594 | 0.207 |
| Poorly Characterized | 65,318 ± 3,873 | 67,917 ± 3,919 | -3.827 | 3.102 | 0.078 |
| Replication and Repair | 80,284 ± 4,807 | 79,728 ± 5,180 | 0.697 | 0.110 | 0.740 |
| Signal Transduction | 28,736 ± 2,430 | 31,462 ± 4,108 | -8.666 | 4.665 | **0.031** |
| Signaling Molecules and Interaction | 2,084 ± 304 | 2,259 ± 521 | -7.735 | 0.487 | 0.485 |
| Transcription | 29,913 ± 1,699 | 31,744 ± 1,774 | -5.767 | 6.718 | **0.010** |
| Translation | 49,828 ± 5,245 | 45,541 ± 3,983 | 9.413 | 4.109 | **0.043** |
| Transport and Catabolism | 4,053 ± 532 | 3,408 ± 639 | 18.900 | 5.884 | **0.015** |
| Xenobiotics Biodegradation and Metabolism | 43,846 ± 9,770 | 36,934 ± 3,530 | 18.712 | 4.244 | **0.039** |
