# Supplementary material for: Characterization of the microbiome of the invasive Asian toad in Madagascar across the expansion range and comparison with a native co-occurring species
Source: PeerJ. 2021 Jun 28;9:e11532. doi: 10.7717/peerj.11532 (PMC8247705; doi:10.7717/peerj.11532)
Supplement: Supplemental Information 9 — Groups that show significant higher abundance levels (Kruskal-Wallis test) in males (green) and females (orange) are highlighted. [file peerj-09-11532-s009.docx]

**Table S4:**

**Predicted abundance of KEGG ortholog groups (Level 2 KOs) from skin bacterial communities of males and females of *Duttaphrynus melanostictus*.**

Groups that show significant higher abundance levels (Kruskal-Wallis test) in males (green) and females (orange) are highlighted.

| **KEGG pathways (Level 2)** | **Males** | **Females** | **% Difference (Males/Females)** | **Kruskal-Wallis** | ***p-value*** |  |
| --- | --- | --- | --- | --- | --- | --- |
| Amino Acid Metabolism | 125,184 ± 4,161 | 121,131 ± 10,025 | 3.346 | 2.344 | 0.126 |  |
| Biosynthesis of Other Secondary Metabolites | 10,371 ± 732 | 10,471 ± 871 | -0.951 | 0.356 | 0.551 |  |
| Cancers | 2,102 ± 354 | 1,877 ± 254 | 12.008 | 4.639 | 0.031 |  |
| Carbohydrate Metabolism | 121,273 ± 3,285 | 120,009 ± 4,485 | 1.054 | 2.506 | 0.113 |  |
| Cardiovascular Diseases | 242 ± 143 | 140 ± 121 | 73.299 | 6.600 | 0.010 |  |
| Cell Growth and Death | 7,005 ± 1,266 | 6,397 ± 1,039 | 9.514 | 1.617 | 0.204 |  |
| Cell Motility | 38,441 ± 5,703 | 45,490 ± 13,819 | -15.495 | 2.845 | 0.092 |  |
| Cellular Processes and Signaling | 48,929 ± 4,809 | 51,919 ± 5,176 | -5.759 | 1.752 | 0.186 |  |
| Circulatory System | 514 ± 69 | 573 ± 226 | -10.343 | 2.673 | 0.102 |  |
| Digestive System | 501 ± 106 | 657 ± 228 | -23.663 | 2.346 | 0.126 |  |
| Endocrine System | 4,402 ± 569 | 4,249 ± 910 | 3.589 | 1.245 | 0.264 |  |
| Energy Metabolism | 63,993 ± 1,613 | 62,047 ± 3,042 | 3.135 | 2.188 | 0.139 |  |
| Environmental Adaptation | 1,735 ± 320 | 2,042 ± 459 | -15.044 | 2.188 | 0.139 |  |
| Enzyme Families | 21,466 ± 1,186 | 21,873 ± 931 | -1.859 | 0.297 | 0.586 |  |
| Excretory System | 394 ± 56 | 343 ± 180 | 14.894 | 2.344 | 0.126 |  |
| Folding, Sorting and Degradation | 26,938 ± 2,089 | 30,001 ± 3,261 | -10.209 | 5.576 | 0.018 |  |
| Genetic Information Processing | 27,673 ± 1,559 | 29,510 ± 2,834 | -6.224 | 3.589 | 0.058 |  |
| Glycan Biosynthesis and Metabolism | 24,040 ± 3,100 | 26,300 ± 3,468 | -8.595 | 2.506 | 0.113 |  |
| Immune System | 651 ± 116 | 725 ± 117 | -10.165 | 3.302 | 0.069 |  |
| Immune System Diseases | 589 ± 112 | 662 ± 70 | -11.064 | 4.531 | 0.033 |  |
| Infectious Diseases | 5,272 ± 454 | 5,642 ± 945 | -6.549 | 1.245 | 0.264 |  |
| Lipid Metabolism | 45,597 ± 2,412 | 43,876 ± 6,101 | 3.923 | 3.023 | 0.082 |  |
| Membrane Transport | 159,364 ± 16,104 | 140,525 ± 18,872 | 13.406 | 6.077 | 0.014 |  |
| Metabolic Diseases | 854 ± 94 | 858 ± 77 | -0.399 | 0.195 | 0.659 |  |
| Metabolism | 32,988 ± 1,552 | 35,060 ± 3,206 | -5.912 | 1.488 | 0.223 |  |
| Metabolism of Cofactors and Vitamins | 48,658 ± 1,733 | 50,750 ± 2,711 | -4.121 | 4.418 | 0.036 |  |
| Metabolism of Other Amino Acids | 24,003 ± 1,399 | 23,005 ± 2,165 | 4.337 | 2.188 | 0.139 |  |
| Metabolism of Terpenoids and Polyketides | 24,779 ± 1,678 | 23,747 ± 3,403 | 4.347 | 2.344 | 0.126 |  |
| Nervous System | 933 ± 143 | 821 ± 233 | 13.655 | 2.037 | 0.154 |  |
| Neurodegenerative Diseases | 4,140 ± 585 | 3,813 ± 848 | 8.562 | 2.506 | 0.113 |  |
| Nucleotide Metabolism | 37,180 ± 1,781 | 38,351 ± 1,970 | -3.053 | 2.673 | 0.102 |  |
| Poorly Characterized | 63,968 ± 2,873 | 67,882 ± 4,133 | -5.766 | 5.097 | 0.024 |  |
| Replication and Repair | 79,284 ± 5,118 | 82,584 ± 3,716 | -3.996 | 3.395 | 0.065 |  |
| Signal Transduction | 28,168 ± 1,478 | 29,813 ± 3,190 | -5.520 | 3.395 | 0.065 |  |
| Signaling Molecules and Interaction | 2,243 ± 198 | 1,911 ± 327 | 17.354 | 6.077 | 0.014 |  |
| Transcription | 30,023 ± 1,841 | 30,173 ± 1,578 | -0.499 | 0.243 | 0.622 |  |
| Translation | 48,067 ± 4,013 | 52,867 ± 5,811 | -9.078 | 3.589 | 0.058 |  |
| Transport and Catabolism | 4,270 ± 374 | 3,823 ± 614 | 11.686 | 3.993 | 0.046 |  |
| Xenobiotics Biodegradation and Metabolism | 46,507 ± 8,030 | 40,828 ± 11,401 | 13.910 | 2.344 | 0.126 |  |
