# Supplementary material for: Characterization of the microbiome of the invasive Asian toad in Madagascar across the expansion range and comparison with a native co-occurring species
Source: PeerJ. 2021 Jun 28;9:e11532. doi: 10.7717/peerj.11532 (PMC8247705; doi:10.7717/peerj.11532)
Supplement: Supplemental Information 10 — Colored in yellow are the groups that were more abundant in D. melanostictus and in blue groups more abundant in P. mascareniensis. [file peerj-09-11532-s010.docx]

**Table S5:**

**Predicted abundance of KEGG ortholog groups (Level 2 KOs) from gut bacterial communities of *Duttaphrynus melanostictus* and *Ptychadena mascareniensis* that present significant difference (Kruskal-Wallis test) in the abundance levels between host species.**

Colored in yellow are the groups that were more abundant in *D. melanostictus* and in blue groups more abundant in *P. mascareniensis*.

| **KEGG Pathways (Level 2)** | ***Duttaphrynus***  ***melanostictus*** | ***Ptychadena***  ***mascareniensis*** | ***% differences D.melanostictus/ P.mascareniensis*** | **Kruskal-Wallis** | ***p-value*** |
| --- | --- | --- | --- | --- | --- |
| Amino Acid Metabolism | 37,788 ± 1,328 | 36,554 ± 1,866 | 3.377 | 1.190 | 0.275 |
| Biosynthesis of Other Secondary Metabolites | 3,643 ± 208 | 2,936 ± 223 | 24.052 | 3.857 | **0.050** |
| Cancers | 472 ± 120 | 417 ± 52 | 13.168 | 0.441 | 0.507 |
| Carbohydrate Metabolism | 43,505 ± 2,604 | 41,519 ± 865 | 4.783 | 1.190 | 0.275 |
| Cardiovascular Diseases | 13 ± 20 | 7 ± 5 | 90.476 | 0.048 | 0.827 |
| Cell Growth and Death | 1,749 ± 99 | 1,672 ± 212 | 4.603 | 0.048 | 0.827 |
| Cell Motility | 9,141 ± 1,370 | 9,081 ± 1,463 | 0.661 | 0.048 | 0.827 |
| Cellular Processes and Signaling | 17,545 ± 1,332 | 17,509 ± 1,404 | 0.209 | 0.048 | 0.827 |
| Circulatory System | 44 ± 48 | 40 ± 50 | 10.833 | 0.048 | 0.827 |
| Digestive System | 135 ± 66 | 134 ± 61 | 0.496 | 0.048 | 0.827 |
| Endocrine System | 1,206 ± 45 | 881 ± 221 | 36.966 | 3.857 | **0.050** |
| Energy Metabolism | 22,065 ± 1,332 | 19,789 ± 987 | 11.499 | 3.857 | **0.050** |
| Environmental Adaptation | 576 ± 71 | 541 ± 63 | 6.404 | 0.429 | 0.513 |
| Enzyme Families | 8,042 ± 219 | 8,337 ± 282 | -3.542 | 1.190 | 0.275 |
| Excretory System | 135 ± 7 | 121 ± 28 | 11.813 | 0.429 | 0.513 |
| Folding, Sorting and Degradation | 9,199 ± 142 | 8,694 ± 351 | 5.800 | 3.857 | **0.050** |
| Genetic Information Processing | 9,928 ± 742 | 11,126 ± 1,438 | -10.767 | 1.190 | 0.275 |
| Glycan Biosynthesis and Metabolism | 9,344 ± 600 | 7,984 ± 1,876 | 17.024 | 0.429 | 0.513 |
| Immune System | 298 ± 39 | 283 ± 5 | 5.176 | 0.441 | 0.507 |
| Immune System Diseases | 205 ± 34 | 253 ± 49 | -18.709 | 2.333 | 0.127 |
| Infectious Diseases | 1,679 ± 142 | 1,816 ± 378 | -7.543 | 0.429 | 0.513 |
| Lipid Metabolism | 12,546 ± 730 | 12,152 ± 1,170 | 3.237 | 0.429 | 0.513 |
| Membrane Transport | 51,135 ± 896 | 55,072 ± 2,465 | -7.148 | 3.857 | **0.050** |
| Metabolic Diseases | 353 ± 22 | 346 ± 63 | 2.019 | 0.048 | 0.827 |
| Metabolism | 11,103 ± 40 | 12,156 ± 962 | -8.660 | 1.190 | 0.275 |
| Metabolism of Cofactors and Vitamins | 16,478 ± 873 | 15,974 ± 621 | 3.155 | 0.429 | 0.513 |
| Metabolism of Other Amino Acids | 6,706 ± 463 | 6,698 ± 285 | 0.129 | 0.048 | 0.827 |
| Metabolism of Terpenoids and Polyketides | 6,667 ± 465 | 6,599 ± 337 | 1.020 | 0.048 | 0.827 |
| Nervous System | 354 ± 36 | 313 ± 34 | 13.071 | 2.333 | 0.127 |
| Neurodegenerative Diseases | 639 ± 328 | 699 ± 147 | -8.671 | 0.048 | 0.827 |
| Nucleotide Metabolism | 14,157 ± 1,106 | 14,572 ± 946 | -2.843 | 1.190 | 0.275 |
| Poorly Characterized | 20,800 ± 386 | 20,894 ± 1,008 | -0.448 | 0.048 | 0.827 |
| Replication and Repair | 29,801 ± 876 | 31,553 ± 1,740 | -5.553 | 1.190 | 0.275 |
| Signal Transduction | 8,432 ± 429 | 7,666 ± 820 | 9.991 | 1.190 | 0.275 |
| Signaling Molecules and Interaction | 848 ± 127 | 892 ± 114 | -4.966 | 0.429 | 0.513 |
| Transcription | 10,431 ± 901 | 11,533 ± 406 | -9.560 | 2.333 | 0.127 |
| Translation | 18,156 ± 1,097 | 18,875 ± 1,377 | -3.813 | 0.429 | 0.513 |
| Transport and Catabolism | 1,184 ± 295 | 910 ± 249 | 30.135 | 1.190 | 0.275 |
| Xenobiotics Biodegradation and Metabolism | 9,731 ± 1,551 | 9,633 ± 1,778 | 1.010 | 0.429 | 0.513 |
