# Supplementary material for: Characterization of the microbiome of the invasive Asian toad in Madagascar across the expansion range and comparison with a native co-occurring species
Source: PeerJ. 2021 Jun 28;9:e11532. doi: 10.7717/peerj.11532 (PMC8247705; doi:10.7717/peerj.11532)
Supplement: Supplemental Information 11 — In accordance with previous studies only results of dataset A (after rarefaction at 4,000 reads) are shown. [file peerj-09-11532-s011.docx]

**Table S6:** **Comparison of average values of bacterial richness (number of OTUs) obtained in this this study with two previous recent studies on Malagasy terrestrial and aquatic amphibians. In accordance with previous studies only results of dataset A (after rarefaction at 4,000 reads) are shown.**

| Number of OTUs | | | |
| --- | --- | --- | --- |
|  | **This study** | **Bletz *et al*., 2017a** | **Kueneman *et al*., 2019 (values retrieved from the plot)** |
| *Duttaphrynus melanostictus* | 235.9 ± 76 |  |  |
| *Ptychadena mascareniensis* | 78.68 ± 53.1 |  |  |
| Terrestrial amphibians (Madagascar, including *P. mascareniensis*) |  | 277.1 ± 13.9 | 240 |
| Aquatic amphibians (Madagascar) |  | 239.4 ± 14.6 | 125 |
